# SGBP-B-like bimodular cellulose-binding protein CHU_1279 is essential for cellulose utilization by *Cytophaga hutchinsonii*

**DOI:** 10.1128/aem.02471-24

**Published:** 2025-03-25

**Authors:** Weixin Zhang, Lizhu Li, Tengxin Li, Xin Li, Xia Wang, Qiang Yao, Xuemei Lu, Guanjun Chen, Weifeng Liu

**Affiliations:** 1 State Key Laboratory of Microbial Technology, Shandong University 214177 , Qingdao, China; 2 National Center of Technology Innovation for Comprehensive Utilization of Saline-Alkali Land, Dongying, China; Shanghai Jiao Tong University, Shanghai, China

**Keywords:** *Cytophaga hutchinsonii*, cellulose utilization, SGBP-B, PUL

## Abstract

The widespread cellulolytic specialist *Cytophaga hutchinsonii* belonging to the phylum Bacteroidetes adopted a unique cellulose utilization strategy that did not conform to the known cellulose-degrading paradigms involving free cellulases or cellulosomes. The strategy used by *C. hutchinsonii* still remains largely unclear. In this study, we showed that *chu_1279* within the *chu_1276–chu_1280* gene cluster, which has been previously shown to be important for cellulose utilization by *C. hutchinsonii*, encodes an outer membrane protein, and its elimination prohibited bacterial growth on cellulose. Structural prediction revealed that CHU_1279 is a surface glycan-binding protein B (SGBP-B)-like protein comprising two putative carbohydrate-binding module (CBM)-like domains. Further analyses verified that recombinant CHU_1279 displayed significant cellulose-binding protein, and its C-terminal domain is predominantly responsible for cellulose binding. Expression of the C-terminal domain but not the N-terminal domain restored cellulose utilization of ∆*chu_1279*. Moreover, site-directed mutagenesis analyses identified three aromatic residues important for cellulose binding of the recombinant CHU_1279 protein. The defective cellulose utilization of ∆*chu_1279* cells otherwise could be recovered by CHU_1279 variants with significantly damaged cellulose-binding capability. Sequence analyses revealed that orthologs of CHU_1279 as well as the atypical polysaccharide utilization loci (PUL) constituted by the gene cluster *chu_1276–chu_1280* are also present in two other cellulolytic Bacteroidetes bacteria, *Cytophaga aurantiaca* and *Sporocytophaga myxococcoides*, which are closely related to *C. hutchinsonii*. Our results contribute to unveiling the unique mechanism underlying the efficient cellulose utilization by *C. hutchinsonii* and similar cellulolytic bacteria.

**IMPORTANCE:**

Most members of the phylum Bacteroidetes are highly competitive and efficient degraders of complex polysaccharides largely ascribed to their employment of a SusC-like system encoded by a polysaccharide utilization locus (PUL). However, characterization of PULs is limited to those responsible for utilization of (semi)soluble glycans. PULs involved in the utilization of cellulose, the most abundant renewable polymer, have not been identified and functionally characterized yet. We demonstrated that *chu_1279* in the cellulolytic specialist *C. hutchinsonii* encodes an SGBP-B-like protein that is required for cellulose utilization, supporting that the gene cluster *chu_1276–chu_1280* in *C. hutchinsonii* encodes an atypical PUL system dedicated to cellulose assimilation. Further analyses showed that this atypical PUL system is also present in two other cellulolytic Bacteroidetes bacteria. This study not only contributes to unveiling the unusual cellulose utilization strategy adopted by *C. hutchinsonii* and similar cellulolytic bacteria but also helps expand our understanding of atypical PULs for nutrient acquisition by cellulolytic bacteria.

## INTRODUCTION

Cellulose is the most abundant and renewable polysaccharide on earth and has great potential for bioconversion applications (1). In nature, a number of aerobic or anaerobic microorganisms are efficient cellulose degraders (2). There are two well-known cellulose-utilizing strategies; one is used by some filamentous fungi and aerobic bacteria that secrete robust extracellular free cellulases (2, 3), and the other by anaerobic bacteria, such as *Clostridium thermocellum*, that form the cell surface-anchored multiprotein called the cellulosome (4, 5). However, a few cellulolytic bacteria, as represented by the widespread cellulolytic specialist *Cytophaga hutchinsonii* belonging to the phylum Bacteroidetes, do not seem to conform to these two cellulose-degrading paradigms. They neither secrete a free cellulase system nor form cellulosomes, but instead digest crystalline cellulose in a substrate contact-dependent manner (6–8). Genomic analysis of *C. hutchinsonii* revealed several unusual features, such as the absence of obvious homologs of cellobiohydrolases, and all annotated endoglucanases lack recognizable carbohydrate-binding modules (CBMs) (6). Up to now, the unique strategy used for efficient cellulose utilization by *C. hutchinsonii* and similar cellulolytic bacteria has not been completely understood.

For cell propagation and colonization, bacteria must rapidly detect extracellular resources and then catabolize them efficiently. Most members of the phylum Bacteroidetes are highly competitive and efficient degraders of complex polysaccharides (9). This ability of Bacteroidetes bacteria is largely ascribed to their employment of a SusC-like system, a protein complex that is localized on the cell surface and acts in concert to capture, degrade, and import the targeted carbohydrates (10–12). The SusC-like system is usually encoded by a polysaccharide utilization locus (PUL), a gene cluster that is transcriptionally activated in response to the polysaccharide substrate (13). A hallmark of canonical PULs is the presence of at least one gene pair of *TBDT*/*SGBP-A* (*susC*/*susD* homolog) encoding a core outer membrane TonB-dependent transporter (TBDT) and a tightly associated cell-surface glycan-binding protein A (SGBP-A), respectively, to form an active transport complex that is crucial for nutrient acquisition (11, 13–15). Besides, PULs typically have one or more additional, nonhomologous SGBP(s) (e.g., SGBP-B), which is also capable of glycan binding and thus may play important roles in assisting polysaccharide capture to the cell surface for assimilation presumably by increasing local substrate concentration (16–20). Unlike SGBPs-A that share significant sequence and structural conservation, SGBPs-B display poor sequence similarities, which may explain for the less successful identification of their homologs by bioinformatics approaches (11, 14). Crystal structural analyses revealed that SGBPs-B are typified by multi-domain architectures, with a significant variation in the domain number (16, 21). This structural feature might be beneficial to increase the flexibility of SGBP-B and therefore promote polysaccharide capture. Despite these advances, characterization of SGBPs hitherto is limited to those from PULs responsible for utilization of (semi)soluble glycans (11), and potential SGBPs involved in utilization of crystalline cellulose have not been identified and functionally characterized yet, which impedes the detailed understanding of the role of specific PULs in mediating cellulose assimilation by cellulolytic Bacteroidetes bacteria.

Whereas genome analysis indicated that two obvious SusC/SusD (TBDT/SGBP-A) homologs are present in *C. hutchinsonii*, neither of them is involved in cellulose utilization (22). On the other hand, previous studies suggested that a gene cluster including *chu_1276–chu_1280* is essentially involved in the cellulolytic process of *C. hutchinsonii* (23–27). These genes are transcribed in the same orientation and activated simultaneously in response to cellulose (23), which are thought to act together in the process of cellulose utilization. Specifically, the gene *chu_1276* encoding an outer membrane protein is required for cellulose degradation by *C. hutchinsonii* (23), and it is the critical regulatory target of an extracytoplasmic function (ECF) σ-factor that controls *C. hutchinsonii* cellulolytic response to cellulose (28). In addition, the deletion of *chu_1277*, which also encodes an outer membrane protein, or *chu_1280* encoding an endoglucanase exerted a marked impact on *C. hutchinsonii* cellulose utilization (26, 27). Given that atypical PULs have been discovered in a few bacterial species (29, 30), it is thus highly possible that the *chu*_*1276–1280* cluster represents a novel type of PUL involved in cellulose utilization (28). However, more experimental data are needed to support this speculation. In particular, the function of two genes within this cluster, *chu_1278* and *chu_1279*, both encoding hypothetical proteins, awaits clarification.

In this study, we investigated the function of *chu_1279* in cellulose utilization by *C. hutchinsonii*. Our data showed that CHU1279 is an outer membrane protein, and its absence prohibited bacterial cellulose assimilation. Further analysis revealed that CHU_1279 is a SGBP-B-like cellulose-binding protein comprising two putative CBM-like domains, and its C-terminal domain is predominantly responsible for cellulose binding. Expression of the C-terminal domain, but not the N-terminal domain, restored cellulose utilization of ∆*chu_1279* cells. Moreover, site-directed mutagenesis analyses identified three aromatic residues important for cellulose binding of the recombinant CHU_1279 protein. The defective cellulose utilization of ∆*chu_1279* cells otherwise could be recovered by CHU_1279 variants with significantly damaged cellulose-binding capability.

## RESULTS

### Deletion of *chu_1279* prohibited cellulose utilization by *C. hutchinsonii*

**Fig 1:**
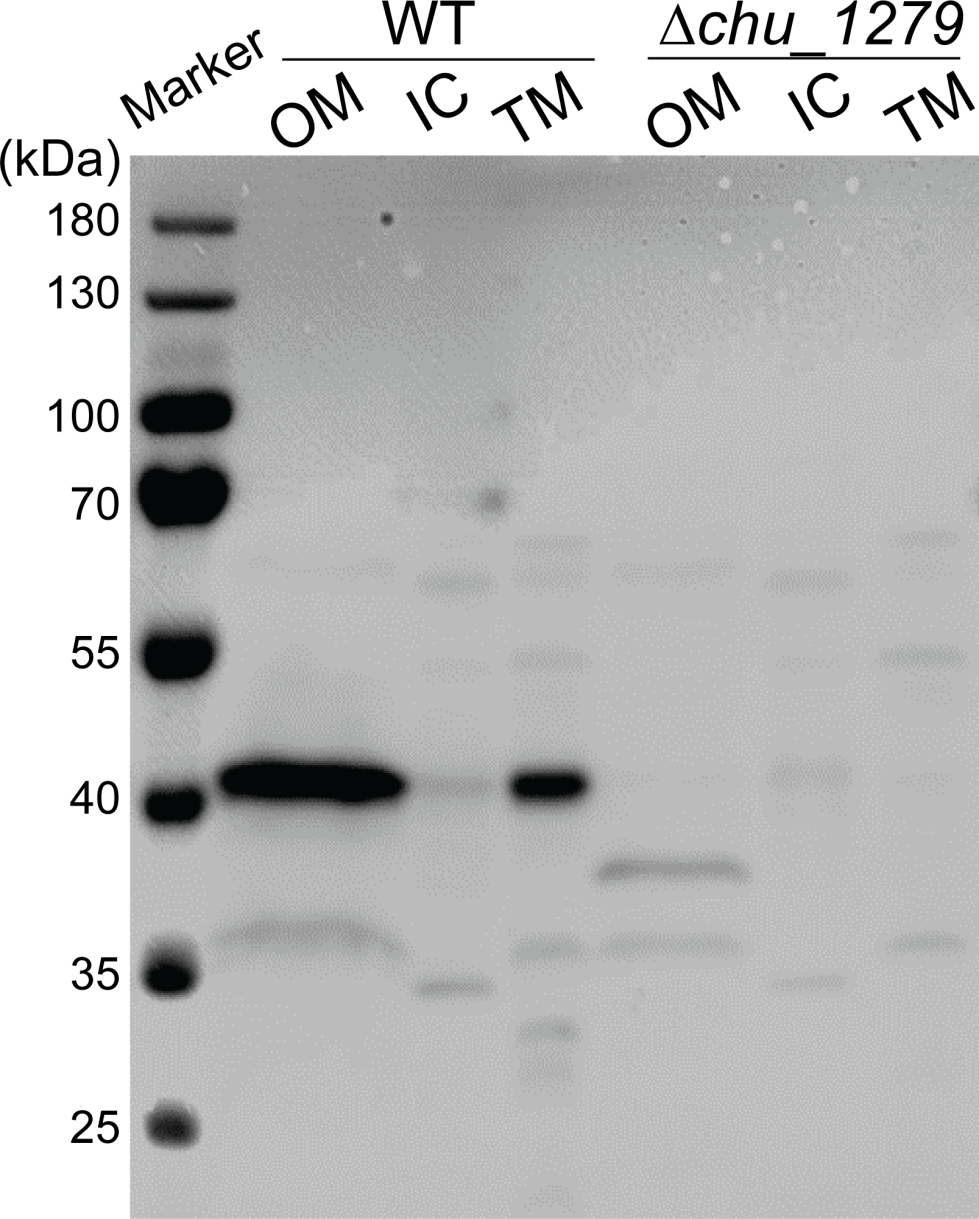
Analysis of subcellular localization of CHU_1279 in *C. hutchinsonii*. WT, wild type strain; ∆*chu_1279, chu_1279* deletion strain; OM, outer membrane fraction; IC, intracellular fraction; TM, total membrane fraction.

Previous results implicated that the gene cluster *chu_1276–chu_1280* may present an atypical PUL essentially involved in cellulose utilization by *C. hutchinsonii* (23, 28). Nonetheless, *chu_1278* and *chu_1279* within this cluster, both encoding hypothetical proteins, have not been functionally characterized regarding their roles in *C. hutchinsonii* cellulose utilization. Here, we focused on *chu_1279* that encodes a 375-aa unknown protein with the first 20 amino acids predicted as a signal peptide. Western blot combined with cellular fractionation analyses showed that CHU_1279 was clearly detected in both fractions of outer membrane and total membrane isolated from the wild-type (WT) cells but was hardly observed in the intracellular protein fraction, indicating that CHU_1279 was located in the outer membrane of *C. hutchinsonii* cells (Fig. 1).

**Fig 2:**
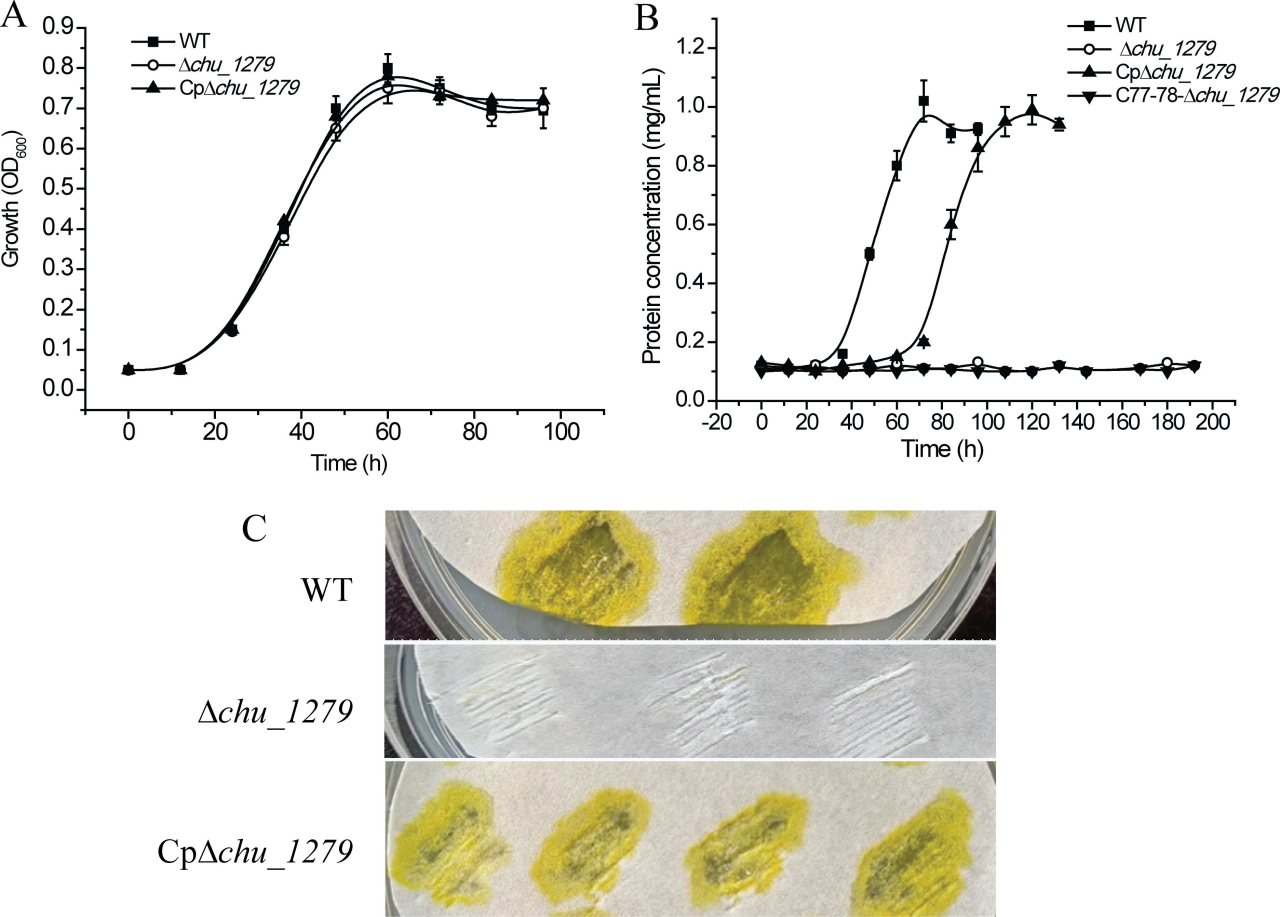
Growth analyses of *C. hutchinsonii* WT and mutant strains with different carbon sources. (A) Growth analyses of WT, ∆*chu_1279*, and Cp∆*chu_1279* in liquid PY10 medium supplemented with 0.4% (wt/vol) glucose. (B) Growth analyses of WT, ∆*chu_1279*, Cp∆*chu_1279*, and C77-78-∆*chu_1279* in liquid PY10 medium supplemented with 2 g/L of Avicel as the sole carbon source. (C) Growth analyses of WT, ∆*chu_1279*, and Cp∆*chu_1279* with filter paper as the sole carbon source for 7 days. WT, wild-type strain; ∆*chu_1279, chu_1279* deletion strain; Cp∆*chu_1279*, ∆*chu_1279* complemented with *chu_1277–chu_1279* driven by the native promoter upstream *chu_1277*; C77-78-∆*chu_1279*, ∆*chu_1279* complemented with *chu_1277–chu_1278* driven by the native promoter upstream *chu_1277*.

**Fig 3:**
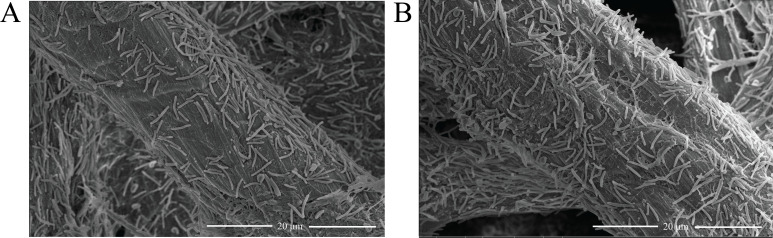
Scanning electron microscopic analysis of the wild-type *C. hutchinsonii* cells (A) and the ∆*chu_1279* cells (B) adhering to filter paper.

**Fig 4:**
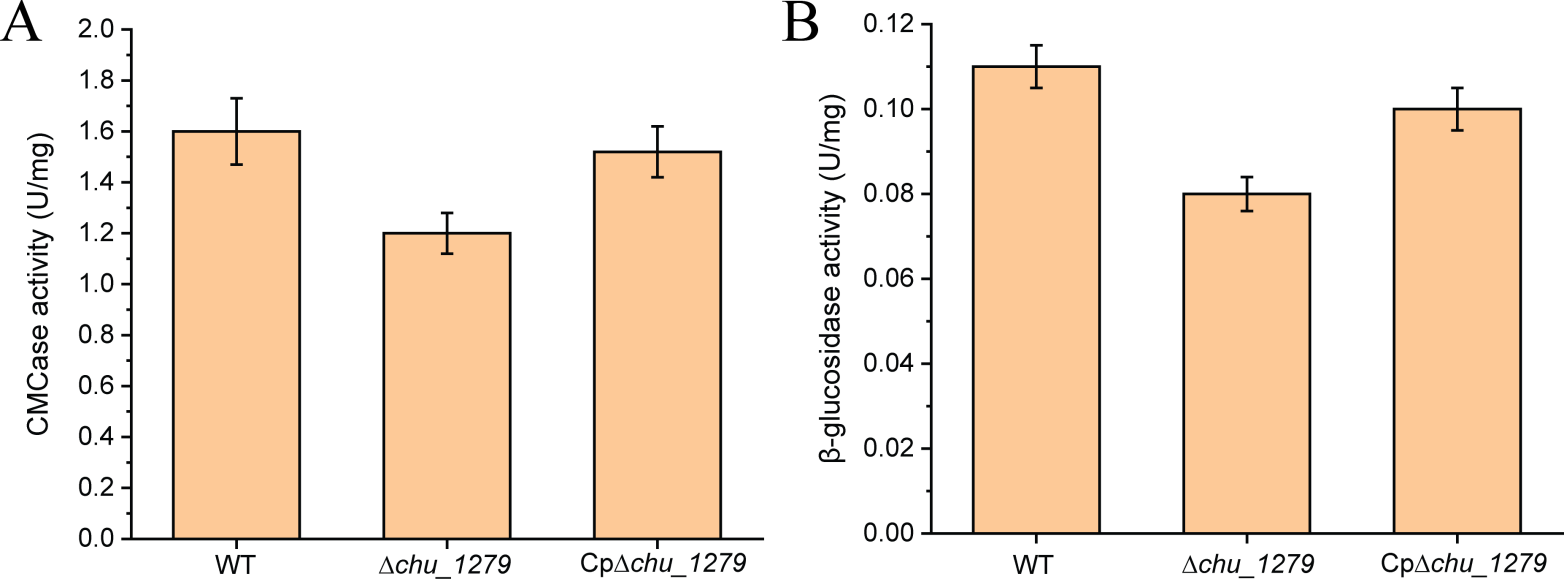
Analyses of endoglucanase (A) and β-glucosidase (B) activities of *C. hutchinsonii* WT, ∆*chu_1279*, and Cp∆*chu_1279* cells. WT, wild-type strain; ∆*chu_1279, chu_1279* deletion strain; Cp∆*chu_1279*, ∆*chu_1279* complemented with *chu_1277–chu_1279* driven by the native promoter upstream *chu_1277*.

Targeted gene deletion of *chu_1279* was then performed, and western blot analysis verified CHU_1279 elimination from not only the outer membrane but also all other cellular fractions in the ∆*chu_1279* mutant (Fig. 1). With 0.4% (wt/vol) glucose or 2 g/L of Avicel cellulose as the sole carbon source, the growth of ∆*chu_1279* was determined. Whereas ∆*chu_1279* grew as well as the WT strain on glucose (Fig. 2A), it was unable to use Avicel even though the cultivation period was extended to 192 h (Fig. 2B). In line with the significant growth defect on Avicel, ∆*chu_1279* did not show growth when incubated on filter paper even after 8 days, and no development of a yellow color as seen associated with the growth of the WT cells was observed with ∆*chu_1279* when grown on filter paper (Fig. 2C). Since adhesion of *C. hutchinsonii* cells to cellulose is considered to be a prerequisite for cellulose degradation (6), the adhering ability of ∆*chu_1279* cells to filter paper was examined by scanning electron microscopic analysis. ∆*chu_1279* cells were densely populated and regularly aligned on the surface of filter paper largely as WT cells did (Fig. 3), indicating that ∆*chu_1279* cells still possessed significant cellulose-adhering capability. Given that *C. hutchinsonii* exhibits cell-associated cellulolytic activities (27), the endoglucanase and β-glucosidase activities of both intact ∆*chu_1279* and WT cells were further measured and compared. As Fig. 4 showed, ∆*chu_1279* cells still displayed obvious cell-associated endoglucanase and β-glucosidase activities despite a slight decrease in ~25% in both activities compared with that of the WT, indicating that the defect in cellulose utilization, as displayed by *chu_1279* deletion may not result from the compromised cellulase activities.

Since genome sequence analysis suggested that *chu_1279* does not have its own promoter, the full nucleotide sequence of *chu_1277–chu_1279*, together with the 123-bp sequence immediately upstream of *chu_1277*, that might act as a weak promoter controlling *chu_1277–chu_1279* expression (23), was introduced into ∆*chu_1279* for complementation (Cp∆*chu_1279*). Meanwhile, a similar complementation plasmid containing only *chu_1277–chu_1278* was introduced into ∆*chu_1279* to generate C77-78-∆*chu_1279* as a control. Growth analyses showed that Cp∆*chu_1279,* but not C77-78-∆*chu_1279* cells, restored typical growth on Avicel or filter paper (Fig. 2), supporting that *chu_1279* is required for cellulose assimilation. Altogether, the above results indicated that the outer membrane protein CHU_1279 plays an essential role in *C. hutchinsonii* cellulose utilization.

### CHU_1279 is comprised of two CBM-like domains and could bind cellulose

**Fig 5:**
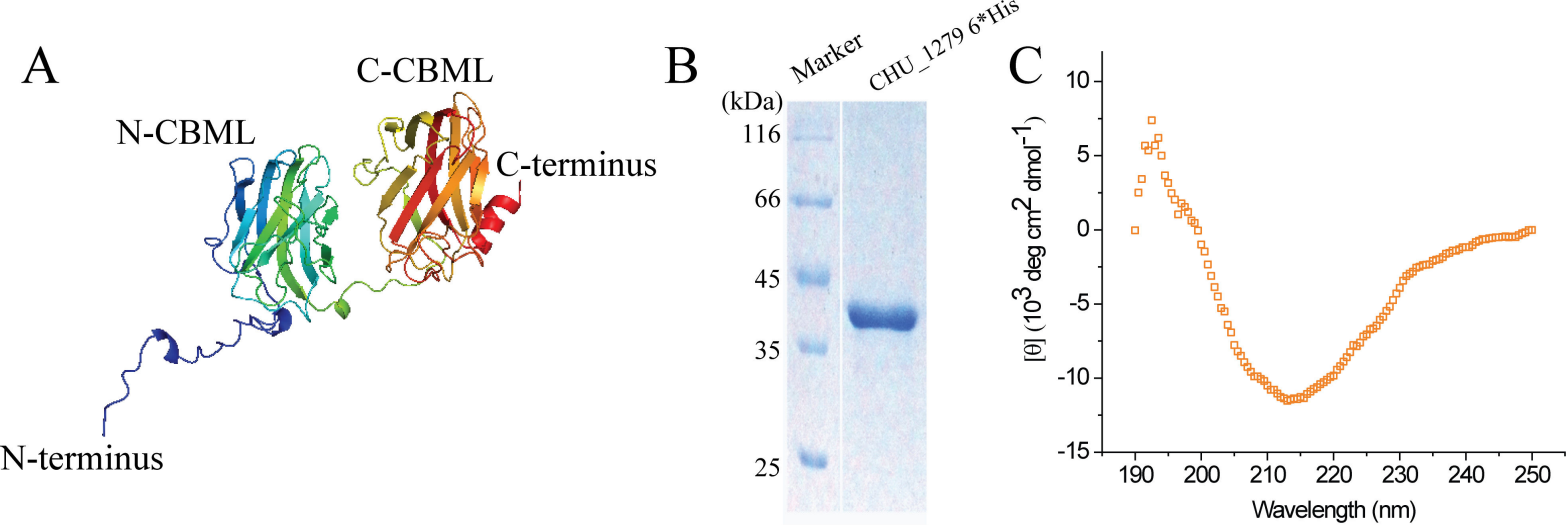
Structural prediction of CHU_1279 and analysis of its secondary structure. (A) Structural prediction of CHU_1279 without the first 20 amino acids using Alpha Fold 2. CHU_1279 contains two CBM-like domains, namely, N-CBML and C-CBML, respectively. (B) SDS-PAGE analysis of purified recombinant CHU_1279 protein from *E. coli* cells tagged with a C-terminal 6*His tag. (C) Circular dichroism spectra profile of the purified recombinant CHU_1279 protein as shown in (B).

**Fig 6:**
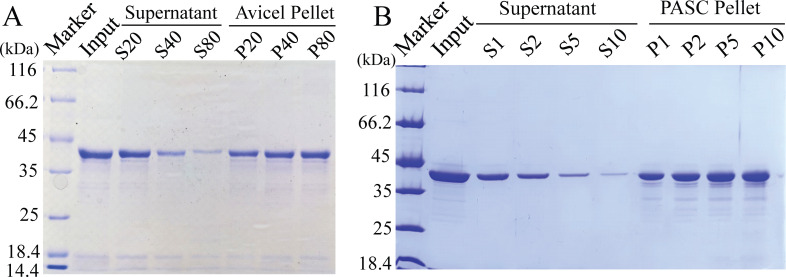
CHU_1279 displayed significant binding ability to cellulose. (A) SDS-PAGE analysis of the supernatant fraction and Avicel fraction after binding assays of CHU_1279 (0.2 mg/mL) with Avicel cellulose. S20, S40, S80: supernatant fraction using 20, 40, and 80 mg of Avicel, respectively. P20, P40, P80: Avicel pellet fraction using 20, 40, and 80 mg of Avicel, respectively. (B) SDS-PAGE analysis of the supernatant fraction and PASC fraction after binding assays of CHU_1279 (0.25 mg/mL) with PASC. S1, S2, S5, S10: supernatant fraction using 1, 2, 5, and 10 mg of PASC, respectively. P1, P2, P5, P10: pellet fraction using 1, 2, 5, and 10 mg of PASC, respectively.

To determine the possible function of CHU_1279, the putative three-dimensional structure of CHU_1279 was determined using Alpha Fold 2. CHU_1279 is composed of two β-sandwich CBM-like domains connected by a disordered linker (Fig. 5A). DALI analysis and HHpred prediction indicated that the overall structure of CHU_1279 is similar to that of CBM-like domain tandem of SGBP-B in β (1, 3)-glucan PUL from *Bacteroides thetaiotaomicron* (16). To experimentally analyze the secondary structure of CHU_1279, CHU_1279, excluding the putative signal peptide but with a 6*His tag, was heterologously expressed and purified from *E. coli* (Fig. 5B). Consistent with the predicted CBM-like structural feature, the circular dichroism profile showed that the recombinant CHU_1279 is typified by β-sandwich folds (Fig. 5C) (31). Given that CBMs are featured by glycan-binding activities, the recombinant CHU_1279 protein was tested for the binding activity to cellulose, the only preferred insoluble carbon source of *C. hutchinsonii*. CHU_1279 exhibited remarkable adhesion to Avicel microcrystalline cellulose, resulting in a significantly decreased level of protein in the supernatant of the binding reactions (Fig. 6A). When increasing amounts of Avicel was used, more CHU_1279 protein was bound to Avicel (Fig. 6A). In addition to Avicel, CHU_1279 also showed obvious binding to phosphoric acid swollen cellulose (PASC) (Fig. 6B), which is considered an amorphous cellulose that is regenerated from Avicel (32). Taken together, these results indicated that CHU_1279 has a significant binding ability to cellulose.

### The C-terminal domain of CHU_1279 is essential for cellulose binding and *C. hutchinsonii* cellulose utilization

**Fig 7:**
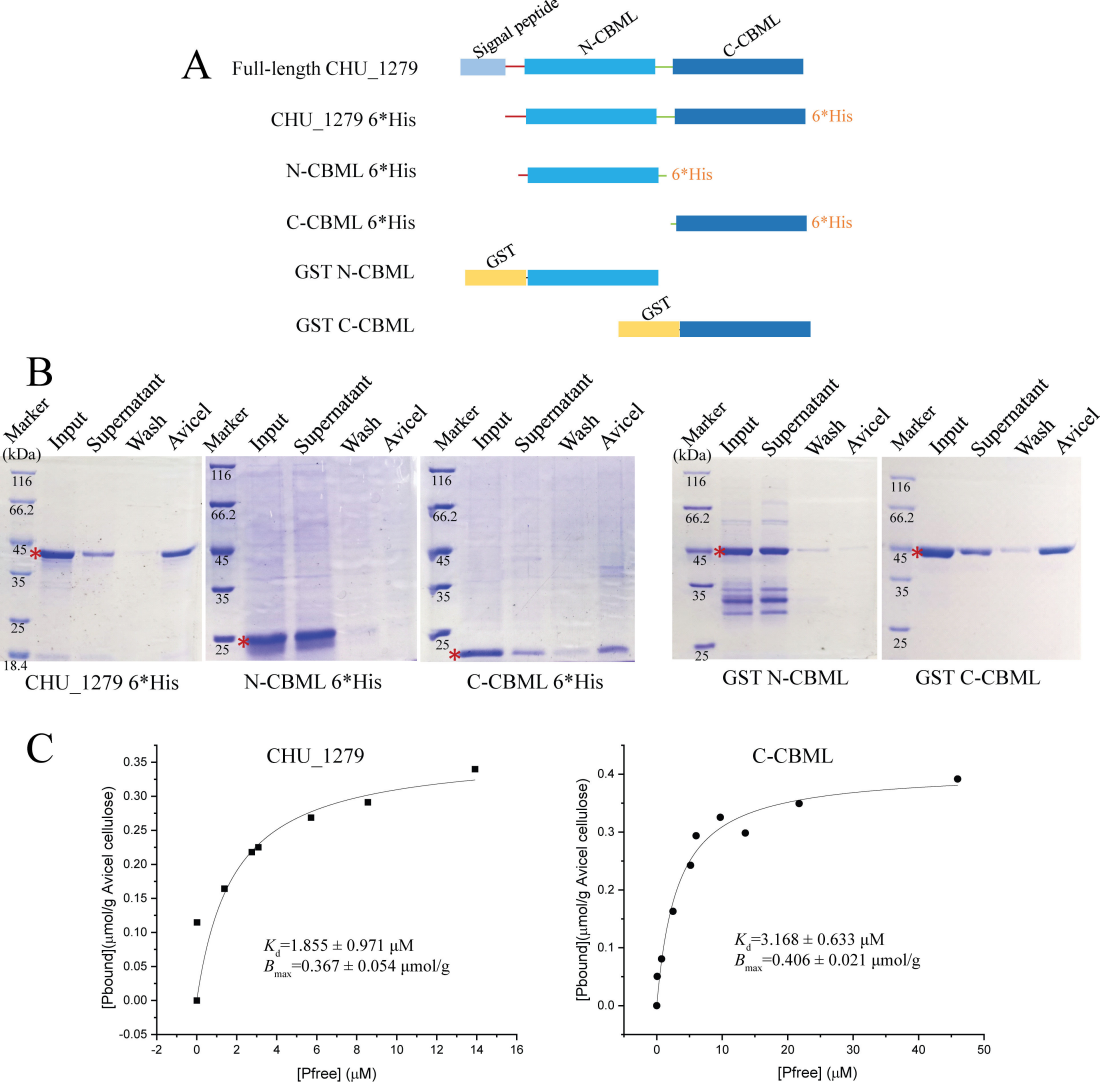
The C-CBML domain is predominantly responsible for cellulose binding by CHU_1279. (A) Schematic illustration of the recombinant protein regions expressed in *E. coli*. (B) Cellulose-binding assays with purified recombinant proteins (0.2 mg/mL) as shown in (A). Fractions obtained in cellulose-binding assays were subjected to SDS-PAGE analyses. Red asterisks indicate the target protein band. (C) The equilibrium adsorption isotherms of full-length CHU_1279 and C-CBML proteins binding to Avicel cellulose.

**Fig 8:**
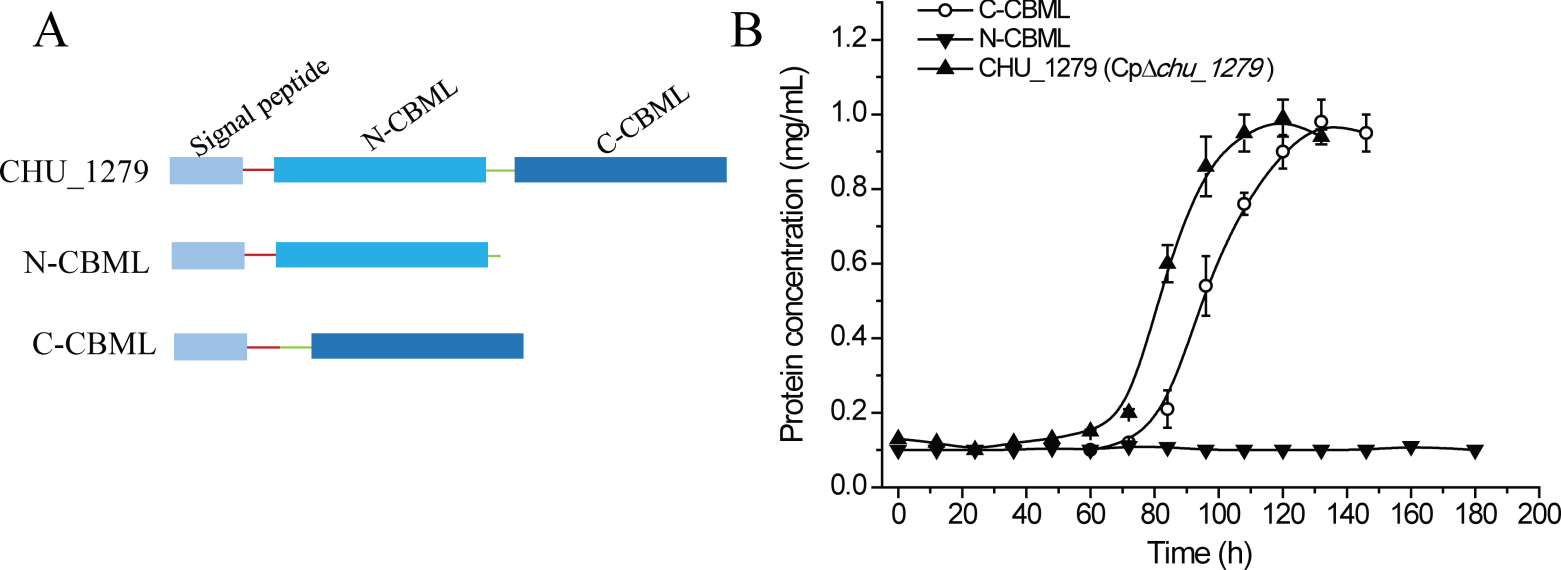
Expression of the C-terminal domain, but not N-terminal domain, restored cellulose utilization of ∆*chu_1279*. (A) Schematic illustration of the CHU_1279 protein regions expressed in ∆*chu_1279*. (B) Growth analyses of ∆*chu_1279* expressing the CHU_1279 variants as shown in (A) with Avicel as the sole carbon source.

To determine the contribution of the two independent CBM-like domains (named N-CBML and C-CBML consisting of Gln48–Glu204 and Glu212–Gln375, respectively) to cellulose binding of CHU_1279, N-CBML and C-CBML were separately expressed and purified from *E. coli* with a C-terminal 6*His tag. Obvious binding to cellulose was observed with C-CBML but not with N-CBML (Fig. 7A and B). To further ascertain this result, each domain was again expressed and purified in *E. coli* with an N-terminal glutathione S-transferase (GST) tag that, itself, has been shown incapable of binding cellulose (33) and subjected to cellulose binding tests. Similar results were observed that C-CBML showed a significant binding ability to cellulose, while hardly any binding was observed when N-CBML was applied (Fig. 7B), demonstrating that the C-terminal CBM-like domain is predominantly responsible for cellulose binding by CHU_1279. To obtain further insight into the cellulose-binding ability between C-CBML and full-length CHU_1279, the equilibrium binding constant (*K*_d_) and maximum binding (*B*_max_) values of the two recombinant proteins with 6*His tag were calculated, respectively. The *K*_d_ and *B*_max_ values obtained were 1.855 ± 0.971 µM and 0.367 ± 0.054 µmol/g for full-length CHU_1279, and 3.168 ± 0.633 µM and 0.406 ± 0.021 µmol/g for C-CBML (Fig. 7C), indicating that, whereas C-CBML displayed a binding capability (*B*_max_) quite similar to that of full-length CHU_1279, it had a slightly lower binding affinity (as shown by *K*_d_) to cellulose than that of full-length CHU_1279. To investigate the *in vivo* role of the two domains in efficient cellulose utilization by *C. hutchinsonii*, N-CBML and C-CBML, directed by the native signal peptide of CHU_1279, were individually expressed in ∆*chu_1279*, using the same strategy for complementation of ∆*chu_1279* with *chu*_1277*–chu*_1279, except that the entire encoding sequence of CHU_1279 was replaced by that of N-CBML or C-CBML (Fig. 8A). Determination of bacterial growth showed that ∆*chu_1279* expressing C-CBML, but not N-CBML, could grow on cellulose, although the growth was delayed by ~12 h compared to that of Cp∆*chu_1279* (Fig. 8B), indicating that C-CBML is the main contributor for the essential role of CHU_1279 in cellulose utilization.

### Identification of key amino acids essential for CHU_1279 binding to cellulose

**Fig 9:**
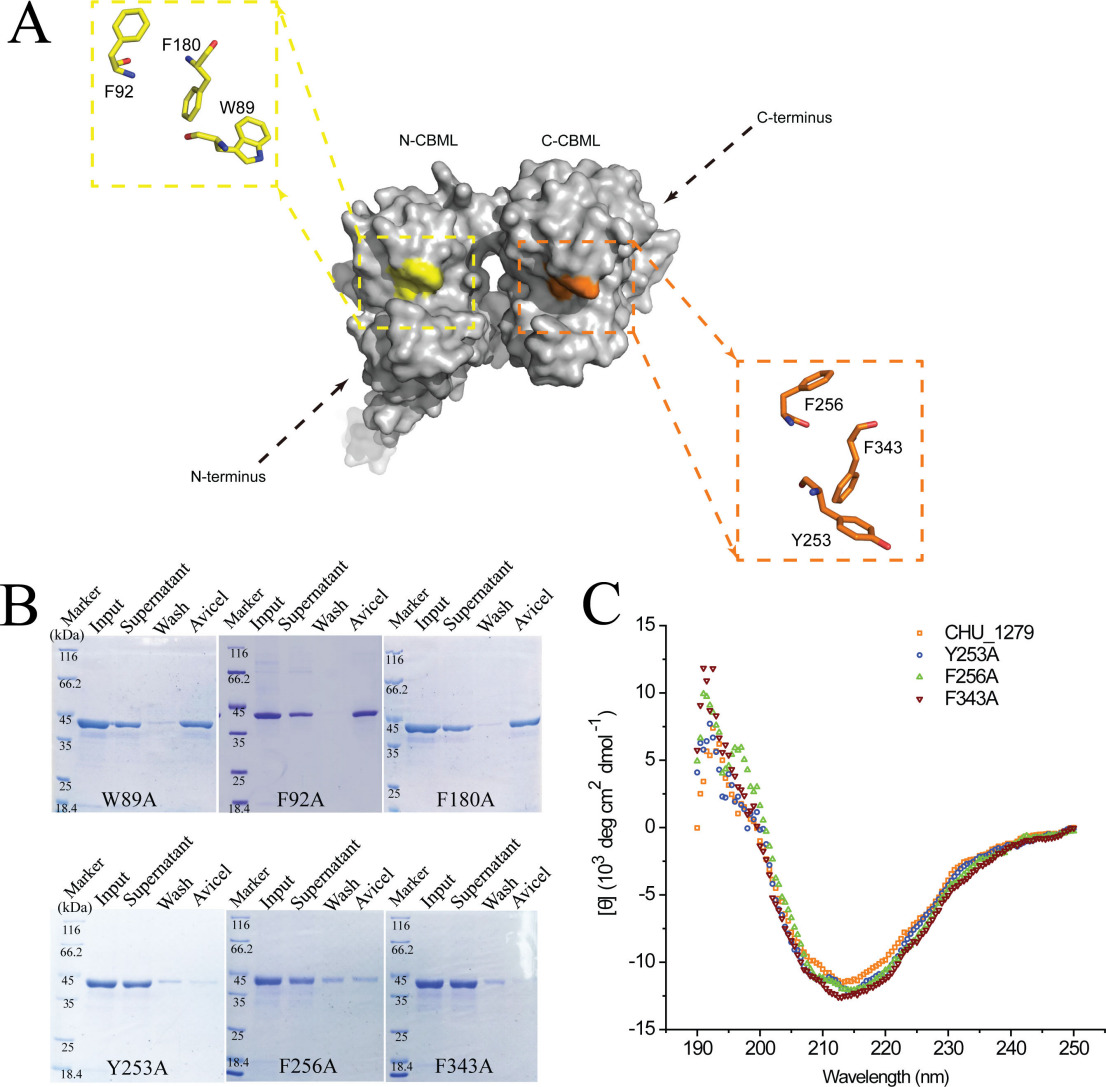
The effect of site-directed mutagenesis on the cellulose-binding ability of CHU_1279. (A) Location of Trp89, Phe 92, Phe180, Tyr 253, Phe 256, and Phe343 in the structure of CHU_1279 obtained using Alpha Fold 2. (B) Cellulose-binding assays with purified recombinant CHU_1279 variants including W89A, F92A, F180A, Y253A, F256A, and F343A. Fractions obtained in cellulose-binding assays with these protein variants were subjected to SDS-PAGE analyses. The concentration of input protein was 0.2 mg/mL. (C) Circular dichroism spectra profiles of the purified recombinant CHU_1279 and its variants Y253A, F256A, and F343A that displayed significantly compromised cellulose-binding ability.

Structural analyses revealed that the atypical tandem of two CBMs similar to that of CHU_1279 was also present in Xyn10C of *Paenibacillus barcinonensis*, a multimodular xylanase with two CBM22s (CBM22-1 and CBM22-2) at its N-terminus to mediate xylan binding (34). Although CHU_1279 has a low sequence identity (~27%) with the CBM tandem of Xyn10C (Fig. S1), sequence alignment revealed that CHU_1279 has equivalent residues identical or similar to three out of six aromatic residues involved in ligand binding in the CBM tandem of Xyn10C, namely, Trp89, Phe180, and Phe343 (Fig. S1). In addition, one more aromatic amino acid Phe92 and two more aromatic amino acids Tyr253 and Phe256 that are located surrounding the putative substrate-binding cleft of N-CBML and C-CBML, respectively (Fig. 9A), were also selected for analysis. Mutations of these six residues were, respectively, performed by replacing each residue with Ala, and the effect on the *in vitro* cellulose binding ability of CHU_1279 was determined (Fig. 9B). The recombinant variants W89A, F92A, and F180A showed similar binding ability to that of wild-type CHU_1279, indicating that Trp89, Phe92, and Phe180 play negligible roles in cellulose binding. In contrast, while mutation of Phe256 significantly decreased CHU_1279 adhesion to cellulose, changes in Tyr253 or Phe343 caused a severe reduction in cellulose binding ability of CHU_1279 (Fig. 9B). A comparison of the circular dichroism (CD) profiles between the wild-type CHU_1279 and the mutant variants Y253A, F256A, or F343A showed that each mutation caused little changes in the overall structure of CHU_1279 (Fig. 9C). The data indicated that the three aromatic residues Tyr253, Phe256, or Phe343 play important roles in the cellulose binding by CHU_1279.

### The essential role of CHU_1279 in *C. hutchinsonii* cellulose utilization extends beyond its ability to bind cellulose

**Fig 10:**
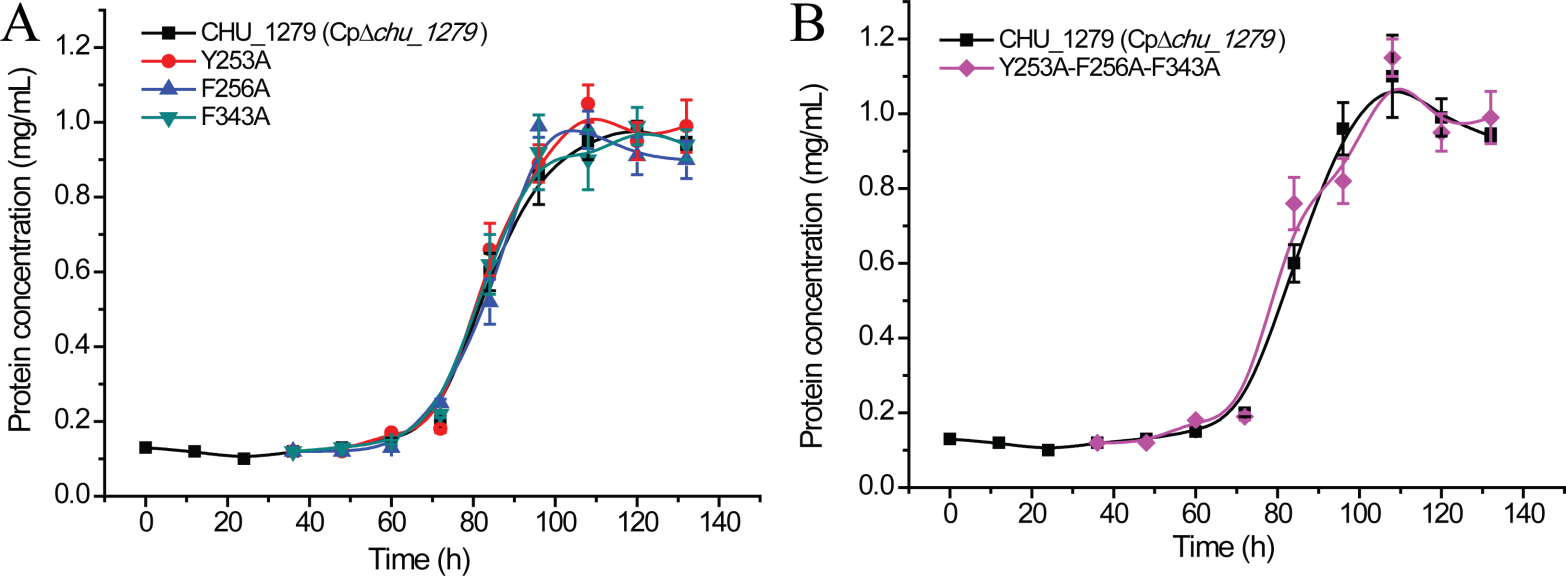
Expression of CHU_1279 variants with significantly compromised cellulose-binding capability could restore cellulose utilization by ∆*chu_1279*. (A) Growth analyses of ∆*chu_1279* expressing the WT CHU_1279 or variants with site-directed mutagenesis (Y253A, F256A or F343A) with Avicel as the sole carbon source. (B) Growth analyses of ∆*chu_1279* expressing the WT CHU_1279 or a triple site-directed mutant (Y253A-F256A-F343A) with Avicel as the sole carbon source.

**Fig 11:**
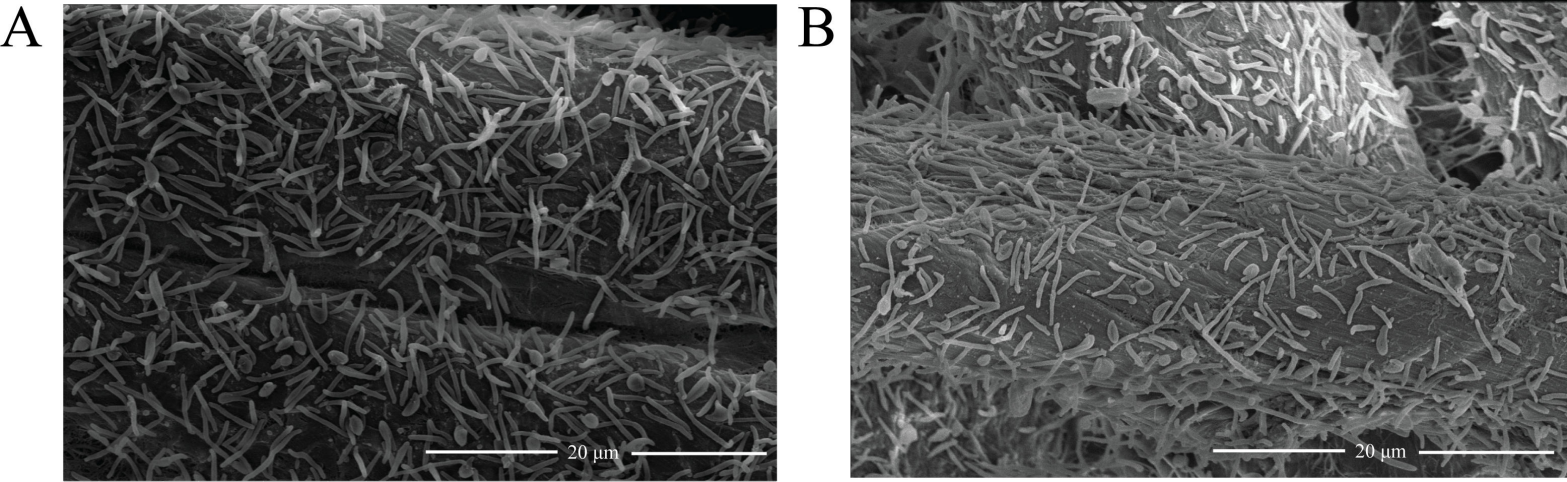
Scanning electron microscopic analysis of *C. hutchinsonii* cells adhering to filter paper. (A) The ∆*chu_1279* cells expressing WT CHU_1279 (Cp∆*chu_1279*); (B) The ∆*chu_1279* cells expressing triple site-directed variant (Y253A-F256A-F343A) of CHU_1279.

Previous reports showed that, whereas the glycan-binding activity is essential to BoSGBP_MLG_-A function for carbohydrate utilization in *B. ovatus* (20), a glycan-binding-deficient variant of SusD or SGBP-A (namely, SusD* and SGBPXyG-A*, respectively) did not compromise the bacterial growth relevant to the corresponding starch assimilation in *B. thetaiotaomicron* or the xyloglucan uptake in *B. ovatus* (17, 35). To investigate whether the identified residues (Tyr253, Phe256, or Phe343) and their mediated cellulose binding play any roles in cellulose utilization by *C. hutchinsonii*, three CHU_1279 variants Y253A, F256A, and F343A were, respectively, expressed in ∆*chu_1279* as described in Materials and Methods, and the growth of the corresponding mutant strains was measured. Results showed that complementation of ∆*chu_1279* with these variants enabled a quite similar bacterial growth on Avicel cellulose to that complemented with the WT CHU_1279 protein (Fig. 10). Similar results were observed when a triple site-directed variant (Y253A-F256A-F343A) of CHU_1279 was introduced into ∆*chu_1279* cells (Fig. 10). Adhesion of cells expressing the triple site-directed variant of CHU_1279 to filter paper was then examined by scanning electron microscopic analysis and compared with that of ∆*chu_1279* cells complemented with WT CHU_1279 (Cp∆*chu_1279*). The overall pattern of adsorption of these two types of cells was quite similar (Fig. 11), indicating that cells expressing the cellulose-binding-deficient variant retained the ability to adhere to cellulose as Cp∆*chu_1279*. Altogether, all these results indicate that the physical presence of CHU_1279 is more critical than its carbohydrate-binding ability for cellulose utilization.

### The atypical PUL is also present in two other cellulolytic Bacteroidetes bacteria closely related to *C. hutchinsonii*

**Fig 12:**
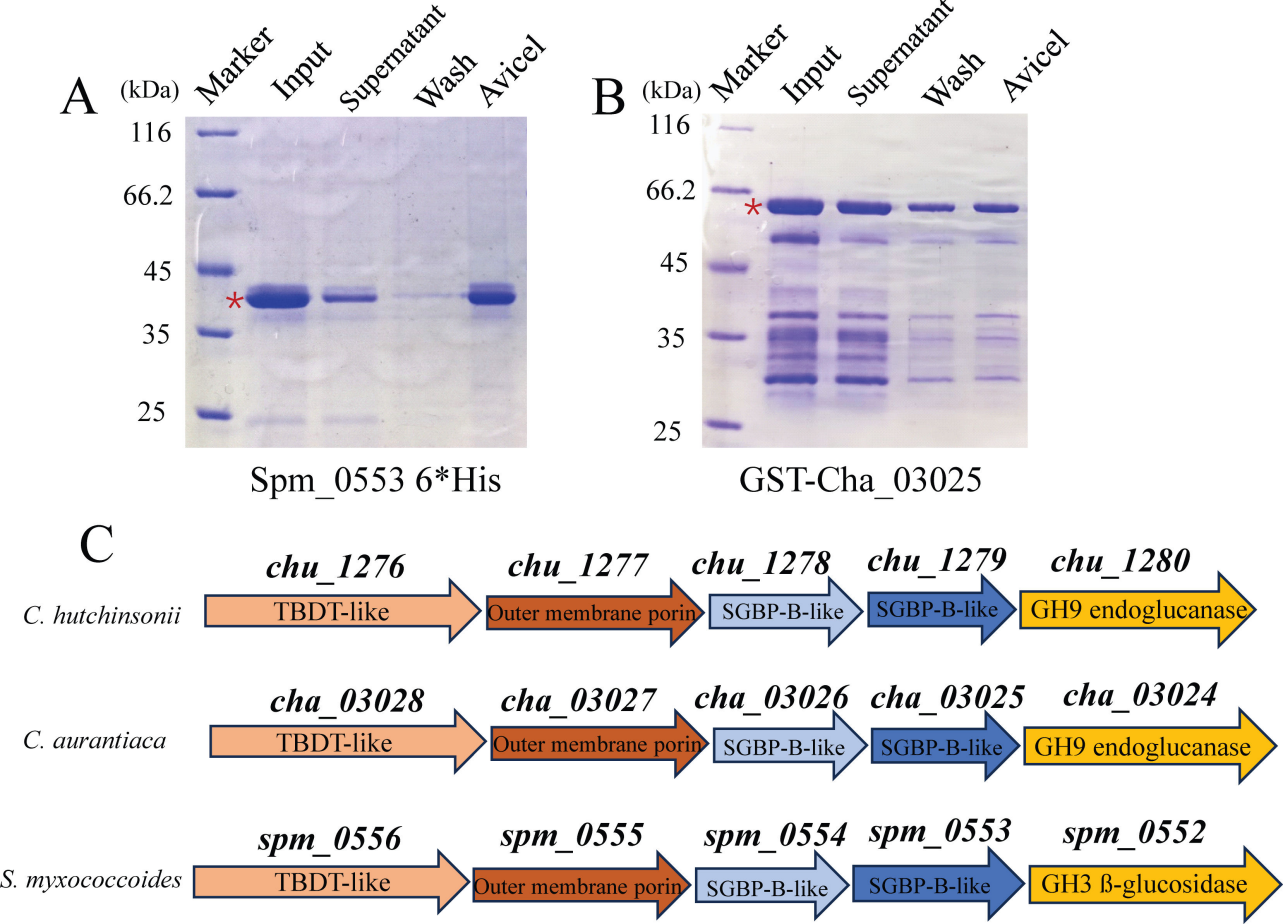
Orthologs of CHU_1279 as well as gene clusters similar to the *chu_1276–chu_1280* cluster of *C. hutchinsonii* were also present in two other cellulolytic bacteria *C. aurantiaca* and *S. myxococcoides*. (A) Cellulose-binding assays with purified recombinant CHU_1279 orthologs in *C. aurantiaca* (A) and *S. myxococcoides* (B). Fractions obtained in cellulose-binding assays with these proteins were subjected to SDS-PAGE analyses. The concentration of input protein was 0.3 mg/mL. Red asterisks indicate the target protein band. (C) Schematic illustration of gene clusters in *C. aurantiaca* and *S. myxococcoides* similar to the *chu_1276–chu_1280* cluster.

To analyze the prevalence of CHU_1279 as well as the involved atypical PUL system for cellulose utilization, the genome-wide searching against the CHU_1279 sequence was performed in two cellulolytic bacteria closely related to *C. hutchinsonii*, *C. aurantiaca*, and *Sporocytophaga myxococcoides* (36). Orthologs of CHU_1279 were present in both bacteria, sharing protein sequence identities of 89% and 49% with CHU_1279, respectively. Neither of them has been previously characterized. Recombinant proteins of these two orthologs exhibited significant cellulose-binding capability as CHU_1279 (Fig. 12A and B). Further analyses of the flanking genomic sequences showed that a similar *chu_1276–chu_1280* cluster was also present in *C. aurantiaca* and *S. myxococcoides* (Fig. 12C), two other cellulolytic bacteria belonging to Bacteroidetes.

## DISCUSSION

The common soil bacterium, *C. hutchinsonii*, is a cellulose-digesting specialist, which has been postulated to use a unique but poorly understood strategy for cellulose utilization (6, 7). *C. hutchinsonii* belongs to the phylum Bacteroidetes, wherein most members employ the PUL-encoding outer membrane SusC-like system for polysaccharide utilization (10, 13). However, systematic analyses have demonstrated that a protein pair homologous to the canonical *B. thetaiotaomicron* SusC/D (TBDT/SGBP-A) proteins are not involved in cellulose utilization by *C. hutchinsonii* (22), leading to the viewpoint that PUL is not used in the process of cellulose digestion (7). Nonetheless, our previous studies suggested that the *chu_1276–1280* cluster may represent a novel type of PUL (23, 28). In this cluster, both *chu_1276* and *chu_1277* encode outer membrane proteins that have been shown to be important for cellulose utilization by *C. hutchinsonii* (23, 26). Structural prediction of CHU_1276 revealed that it probably adopts a β-barrel-like structure, which retrieved several hits to TBDTs, a distinct characteristic of PUL-encoded SusC-like systems. In addition, *chu_1280* encodes an endoglucanase, a kind of hydrolase dedicated to cellulose degradation (25, 27). The presence of a hydrolase for glycan degradation is another common feature observed in PUL systems (10).

Whereas BlastP analysis of the protein sequence showed that CHU_1279 is a hypothetical protein with unknown function, structural prediction using AlphaFold and HHpred suggested that the overall architecture of CHU_1279 is similar to that of the CBM-like domain tandem of SGBP-B in the β (1, 3)-glucan PUL from *B. thetaiotaomicron* (16). We further showed that CHU_1279 is an outer membrane protein with significant cellulose-binding ability, which conformed to the subcellular localization and glycan-binding features of SGBPs in PULs. Based on these analyses, it is reasonable to speculate that CHU_1279 is an SGBP-B-like protein. Structural prediction of CHU_1278 showed that it also has a biomodular β-sandwich architecture similar to the N-terminal two-tandem domains of SGBP-B from *Prevotella bryantii* (PDB access NO. 6D2Y) (unpublished data), implying that CHU_1278 probably represents another SGBP in the PUL system. Unfortunately, since heterologous expression of CHU_1278 in *E. coli* failed to generate soluble active recombinant protein, we were unable to further characterize CHU_1278 (data not shown). Regardless of this, all the above analyses support that the *chu_1276–1280* cluster encodes a novel type of PUL system for cellulose utilization.

Although CHU_1279 has two similar CBM-like domains, our results showed that the C-CBML domain is predominantly responsible for cellulose binding by CHU_1279. Whereas the fold of C-CBML is more similar to that of CBM family 11 (a CBM from *Clostridium thermocellum*, PDB NO. 6R3M), the poor sequence identity with CBM11 members precludes its inclusion in this family. It is possible that C-CBML belongs to a new CBM family, which still needs further exploration for more evidence. Unlike C-CBML, it was found that the N-CBML domain does not display obvious binding to cellulose *in vitro*. Nonetheless, N-CBML should play a role in maintaining the function of CHU_1279 given that while ∆*chu_1279* expressing the triple site-directed variant (W89A-F92A-F180A) showed quite similar growth to that of Cp∆*chu_1279* (expressing WT CHU_1279) on cellulose (Fig. S2), the growth of ∆*chu_1279* expressing C-CBML alone was delayed by ~12 h. Considering that a long polypeptide linker exists between N-CBML and C-CBML domains, it was speculated that the presence of N-CBML and the linker contribute to increase the flexibility of CHU_1279 protein to promote cellulose capture to the bacterial cell surface. Moreover, as reported in other SGBP-Bs (16), the flexibility of the linker may allow different relative conformations of the two CBM-like domains to form the optimal overall architecture for cellulose capture. This may explain our result that the full-length CHU_1279 had a relatively higher binding affinity to cellulose compared with the single C-CBML domain.

In typical PUL systems containing at least one pair of TBDT/SGBP-A (SusD) that is crucial for nutrient acquisition, SGBP-A, but not additional SGBPs (e.g., SGBP-B), is required for glycan utilization. Here in the *chu_1276–1280* cluster, wherein the SGBP-A homolog is lacking, the SGBP-B-like CHU_1279 is required for cellulose utilization. Our results further showed that the physical presence of CHU_1279 is more critical than its cellulose-binding capability for cellulose assimilation, since expression of CHU_1279 variants with significantly reduced cellulose-binding ability recovered the growth of ∆*chu_1279* on cellulose. Similar results were observed for SGBP-A (SusD) homologs in *B. thetaiotaomicron* Sus and *B. ovatus* XyGUL systems (17, 35). Given the fact that SusD directly interacts with SusC to form a complex for nutrient acquisition (15), it is reasonable to speculate that the physical presence of CHU_1279 in *C. hutchinsonii* may help stabilize other components encoded by the *chu_1276–1280* cluster, including CHU_1276 or CHU_1277, or otherwise contribute to the assembly of a larger complex with the rest of the components in the PUL system that is dedicated for cellulose assimilation. Therefore, even with the cellulose-binding-deficient variant (Y253A-F256A-F343A) of CHU_1279, could the whole PUL system still function normally to enable the effective cellulose utilization of *C. hutchinsonii* cells? When CHU_1279 was absent, the stability of other components of the PUL system might be compromised, or the putative larger complex involving other PUL components could not be appropriately assembled to function, and therefore, the cells failed to grow on Avicel cellulose or filter paper, even though the overall cell adhesion to cellulose was not compromised. Considering that the expression of the C-CBML domain of CHU_1279 alone could largely restore the growth defect of ∆*chu_1279* on cellulose, it may well represent a key region involved in such protein interactions or complex assembly. Although the N-CBML domain may not directly participate in the above-conjectured interactions, it is possible that N-CBML functions as a scaffold domain to facilitate the action of C-CBML or the assembly of the putative whole PUL complex. Moreover, we found that ∆*chu_1279* exhibited a remarkable growth defect on glucose at a low concentration of 0.1% (Fig. S3), which was also observed in a mutant strain disrupted for *chu_1276* (23), although the defect in the latter mutant is more severe, suggesting that the absence of CHU_1279 partially interrupts the function of CHU_1276. Another possibility is that CHU_1279 may act together with CHU_1278 to facilitate anchoring of cellulose chains or the intermediate oligosaccharides for further assimilation. In such a way, the complete absence of either CHU_1279 or CHU_1278 would indirectly compromise each other’s function and thus cellulose digestion. Whether CHU_1279 and CHU_1278 fulfill distinctive or cooperative roles in efficient cellulose utilization needs further investigation.

## MATERIALS AND METHODS

### Bacterial strains and culture conditions

*C. hutchinsonii* ATCC 33406, a gift from Dr. MJ McBride at the University of Wisconsin—Milwaukee, was maintained as a glycerol stock in a −80°C freezer in the lab. *C. hutchinsonii* ATCC 33406 was used as the wild-type strain throughout the study. *C. hutchinsonii* cells were grown at 28°C on a rotary shaker (200 rpm) in liquid PY10 medium containing 1% (wt/vol) peptone, 0.05% (wt/vol) yeast extract (pH adjusted to 7.3), and supplemented with different carbon sources including 0.4% (wt/vol) glucose and 2 g/L of Avicel. *Escherichia coli* DH5α was routinely cultured at 37°C in Luria–Bertani medium. Antibiotics were used when necessary at the following concentrations: ampicillin, 100 µg/mL; erythromycin, 60 µg/mL; and chloramphenicol, 15 µg/mL.

### Construction of ∆*chu_1279* and Cp∆*chu_1279*

A 2-kb nucleotide sequence immediately upstream of the start codon of the *chu_1279* gene and a 1.9-kb nucleotide sequence immediately downstream of the stop codon of the *chu_1279* gene were amplified from *C. hutchinsonii* genomic DNA and ligated into the plasmid pYT313 (37), which was transformed into the *C. hutchinsonii* WT cells using electroporation, as previously described (38). Following electroporation, cells were incubated on PY10 agar with glucose and erythromycin at 28°C for 7 days. For SacB-based deletion of *chu_1279*, the erythromycin-resistant cells were transferred to PY10 medium containing glucose but without any antibiotics. The propagated cells were harvested, diluted, and spread on PY10 agar plate with 0.4% (wt/vol) glucose and 5% (wt/vol) sucrose, followed by incubation at 28°C for 5–6 days. Subsequent genomic DNA extraction and anchored PCR were performed to verify the correct integration events in ∆*chu_1279* colonies.

The full nucleotide sequence of *chu_1277–chu_1279*, together with the 123-bp sequence immediately upstream of *chu_1277*, which might act as a weak promoter controlling *chu_1277–chu_1279* expression (23), was amplified from the *C. hutchinsonii* genomic DNA and ligated into pCH03C (23) to construct pCH03C77-79. The resultant plasmid was transformed into ∆*chu_1279* to construct Cp∆*chu_1279*. A similar complementation plasmid containing *chu_1277–chu_1278* and the 123-bp sequence immediately upstream of *chu_1277* was introduced into ∆*chu_1279* to generate C77-78-∆*chu_1279* as a control.

### Construction of *C. hutchinsonii* strains expressing CHU_1279 variants

To express N-CBML of CHU_1279 alone in ∆*chu_1279*, the DNA fragment containing the 123-bp sequence immediately upstream of *chu_1277*, the full nucleotide sequence of *chu_1277–chu_1278*, and the first 636-bp nucleotide acids of the *chu_1279* gene was amplified from *C. hutchinsonii* genomic DNA with a stop codon TAA, followed by ligation into pCH03C to construct pCH03C–N-CBML. To express C-CBML of CHU_1279 alone in ∆*chu_1279*, the DNA fragment containing the 123-bp sequence immediately upstream of *chu_1277*, the full nucleotide sequence of *chu_1277–chu_1278*, and the first 117-bp nucleotide acids of the *chu_1279* gene was amplified from *C. hutchinsonii* genomic DNA and fused together with the last 519-bp nucleotide acids using overlap extension PCR (39). The resultant fragment was ligated into the *Bam*HI/*Xba*I site of pCH03C to yield pCH03C–C-CBML. The plasmids pCH03C–N-CBML and pCH03C–C-CBML were transformed into ∆*chu_1279*, respectively, to construct *C. hutchinsonii* mutant strains expressing CHU_1279 N-CBML or C-CBML.

To express CHU_1279 variants with specific site-directed mutations, overlap extension PCR was performed with pCH03C77-79 as the template. The mutated fragments were ligated into pCH03C, respectively. DNA sequencing was performed to confirm that each mutagenesis occurred as expected. The resultant plasmids were transformed into ∆*chu_1279*, respectively, to generate the *C. hutchinsonii* mutant strains expressing the indicated CHU_1279 variants.

### Growth analysis with different carbon sources

For growth analyses, *C. hutchinsonii* cells were pre-cultured in PY10 medium supplemented with 0.4% (wt/vol) glucose. The cells were collected, washed twice, and transferred to fresh PY10 medium containing the indicated carbon sources. When 0.4% (wt/vol) glucose or 0.1% (wt/vol) glucose was used as a carbon source, the growth of *C. hutchinsonii* was monitored by recording the optical density at 600 nm. When 2 g/L of crystalline cellulose was used, total proteins, which reflected cellular growth, were determined as previously described (40). For the growth assays with filter paper as a carbon source, cells were cultivated until exponential phase in liquid PY10 medium with 0.4% (wt/vol) glucose, collected, and washed with PY10 medium without any carbon sources. Equivalent amounts of *C. hutchinsonii* cells were transferred to PY10 plate with filter paper on top of the agar, and the plate was incubated at 28°C.

### Analysis of subcellular location of CHU_1279 in *C. hutchinsonii*

The total membrane proteins and intracellular soluble proteins were essentially prepared as described by Zhou et al. (41). Briefly, cells were cultivated in PY10 medium with 0.4% (wt/vol) glucose to mid-exponential phase by growth analyses via measurement of optimal density at 600 nm, collected, washed, and disrupted by sonication. The cell debris was removed by centrifugation at 12,000 × *g* for 20 min at 4°C. The supernatant was ultra-centrifuged at 100,000 × *g* for 30 min to obtain the resultant supernatant as intracellular soluble protein fraction. The sediment was resuspended in 50 mM piperazine-*N*,*N*′*-bis* (2-ethanesulfonic acid) (PIPES) buffer (pH 6.8) and used as the total membrane protein fraction. Outer membranes were prepared as previously described by Ji et al. (26, 42). Briefly, cells were collected, washed, and resuspended in PIPES buffer with 0.5 M NaCl, and then incubated at 4°C for 15 min with shaking at 150 rpm, followed by centrifugation at 12,000 × *g* for 20 min to collect supernatant fraction. The supernatant fraction was applied to ultracentrifugation at 100,000 × *g* for 1 h to sedimentate outer membranes. Equal amounts of outer membrane proteins from WT and mutant strains were subject to SDS-PAGE analysis. SDS-PAGE and western blot were performed according to standard protocols. The polyclonal antibody of CHU_1279 used in western blot was prepared by GenScript Biotech Corporation (Nanjing, China). The CHU_1279 peptide of KDINQMDANADSPK was used as the antigen. Immunization of rabbit with the synthesized peptide and subsequent antibody purification was performed according to standard methods.

### Enzymatic assays

*C. hutchinsonii* cells were cultured in PY10 medium with 0.4% (wt/vol) glucose to mid-exponential phase, collected by centrifugation at 5,000 × *g* for 10 min, and then resuspended in 50 mM PIPES buffer at pH 6.8. The endoglucanase and β-glucosidase activities of intact cells were determined as previously described (23), using sodium carboxymethyl cellulose (CMC-Na, Sigma-Aldrich, USA) and then *p*-nitrophenyl β-D-glucopyranoside (*p*NPG, Sigma-Aldrich, USA) as the substrates. One unit of CMCase activity or β-glucosidase activity was defined as the amount of the enzyme releasing 1 µmol of glucose or *p*NP per minute. To measure total protein concentration, cells were washed and resuspended in 0.2 M NaOH, followed by boiling for 20 min. Protein concentration was determined using the BCA method with albumin from bovine serum (BSA) used as standard. Specific activities were represented as U/mg of protein.

### Heterologous expression and purification of CHU_1279 and its variants in *E. coli*

The full-length coding sequence of *chu_1279* excluded with the first 60-bp nucleic acids encoding the putative signal peptide was amplified from the *C. hutchinsonii* genome and inserted into the *Nde*І/*Xho*І site pET22b(+) to construct the plasmid pET-1279. To express CHU_1279 variants with specific site-directed mutations, overlap extension PCR was performed with pET-1279 as the template, and the mutated fragments were ligated into pET22b(+), respectively. The sequences encoding N-CBML and C-CBML were amplified and ligated into pET22b(+) to construct pET–N-CBML and pET–C-CBML. Similarly, the sequences encoding N-CBML and C-CBML were amplified and ligated into the *Bam*HІ/*Xho*І site of pGEX-4T-1, which contains a coding sequence for GST tag, to construct pGEX-N-CBML and pGEX-C-CBML, respectively. To express the CHU_1279 ortholog from *C. aurantiaca*, the coding sequence of the gene *cha_03025* excluded with the first 75-bp nucleic acids encoding the putative signal peptide, was synthesized by GenScript Biotech Corporation (Suzhou, China) and inserted into pGEX-4T-1 to construct the plasmid pGEX-03025. To express the CHU_1279 ortholog from *S. myxococcoides*, the DNA sequence coding for the first 377 amino acids of Spm_0553, but excluded with the first 60-bp nucleic acids encoding the putative signal peptide, was synthesized and ligated into the *Nde*І/*Xho*І site of pET22b(+) to construct the plasmid pET-0553. All the constructed plasmids were individually transformed into *E. coli* BL21 (DE3). *E. coli* transformants were grown at 37°C until the OD_600_ reached 0.5–0.6. IPTG (isopropyl β-D-thiogalactopyranoside) was added at a final concentration of 0.5 mM, and the incubation was continued at 20°C for 16 h. Cells were collected and broken by sonication. Induced recombinant proteins were then purified using affinity chromatography against His-tag or GST-tag, following the manufacturer’s instructions.

### Cellulose-binding assays

Avicel microcrystalline cellulose and PASC were used for cellulose-binding assays. PASC was prepared as previously described by Zhu et al. (40). Protein was incubated with Avicel (PH-101) or PASC in a final volume of 1 mL. For WT CHU_1279 protein, a series of different amounts of Avicel (20, 40, and 80 mg) or PASC (1, 2, 5, and 10 mg) were applied, respectively. For CHU_1279 variants, 20 mg of Avicel was used for assays. Reaction mixtures were incubated at room temperature with rotation for 1 h, and then the pellets were harvested by centrifugation at 2,000 × *g* for 10 min. The supernatant of each sample was collected, and the Avicel or PASC pellet was washed three times with 1 mL of phosphate-buffered saline at pH 7.4, and the first eluent was collected as the wash fraction. The Avicel or PASC was finally resuspended in 1 mL of the same buffer and subjected to SDS-PAGE analyses as the pellet fraction.

### Adsorption isotherms for determination of dissociation constants

The equilibrium binding constant (*K*_*d*_) and binding capacity (*B*_max_) were determined for full-length CHU_1279 and C-CBML domain with a 6*His tag at the C-terminus as previously described (43). Five hundred microliters of CHU_1279 protein solution (0, 45, 120, 200, 215, 340, 460, and 700 µg/mL) and C-CBML protein solution (0, 10, 30, 75, 120, 170, 240, 305, 450, and 930 µg/mL) were mixed with 5 mg of Avicel cellulose, respectively. Reaction mixtures were incubated at room temperature with rotation for 2 h, and then, the pellets were harvested by centrifugation at 8,000 × *g* for 10 min. The supernatant of each sample was collected and subjected to protein concentration quantification using the BCA method. The *K*_*d*_ and *B*_max_ values were determined by fitting the binding isotherms to the equation of [*P*_bound_] = *B*_max_ [*P*_free_]/ *K*_*d*_ + [*P*_free_] by nonlinear regression using the OriginLab software. In the equation, [*P*_free_] represents the supernatant protein concentration after binding, [*P*_bound_] represents bound protein, which equals the initial protein concentration minus [*P*_free_].

### Electron microscopic analysis

*C. hutchinsonii* cells were spotted onto filter paper on top of PY10 agar and incubated at 28°C for 40 min. The filter paper was then washed with 50 mM phosphate buffered saline (PBS) buffer at pH 7.2 for 30 min. The cells that adhered to filter paper were fixed with 2.5% glutaraldehyde in 100 mM PBS buffer at 4°C for 12 h. After being washed with 50 mM PBS buffer three times, the filter paper was dehydrated with increasing concentrations (30%, 50%, 70%, 80%, 90%) of aqueous solutions of ethanol, and finally with 100% ethanol. Samples were mounted, sputter coated with 60% gold and 40% palladium, and finally viewed with a Quanta 250 FEG scanning electron microscope.

### Circular dichroism spectra analyses

CD spectra of CHU_1279 and its variants with the same concentration (0.3 mg/mL) were collected from 260 to 190 nm at a scan speed of 200 nm/min with a bandwidth of 2 nm on a Jasco J-810 spectropolarimeter (Jasco, Japan).

### Sequence analyses

The nucleotide sequences of *chu_1277–chu_1279* were obtained from the KEGG database. Sequences of CHU_1279 orthologs were obtained from the National Center for Biotechnology Information database. Genomic sequences of *C. aurantiaca* and *S. myxococcoides* were obtained from the Joint Genome Institute database. Amino acid sequence alignment was performed using ClustalW (44). N-terminal signal peptide of CHU_1279 was predicted by SignalP (http://www.cbs.dtu.dk/services/SignalP/). The predicted structure of CHU_1276–CHU_1279 proteins was retrieved from the AlphaFold Protein Structure Database. HHpred analyses of protein sequences were undertaken using the server available online (https://toolkit.tuebingen.mpg.de) (45).

## Data Availability

All data generated or analyzed during this study are included in this published article and its supplemental material.

## SUPPLEMENTAL MATERIAL

Supplemental figures aem.02471-24-s0001.docx Figures S1 to S3.

ASM does not own the copyrights to Supplemental Material that may be linked to, or accessed through, an article. The authors have granted ASM a non-exclusive, world-wide license to publish the Supplemental Material files. Please contact the corresponding author directly for reuse.

## References

[1] He ZJ, Chen K, Liu ZH, Li BZ, Yuan YJ. 2023. Valorizing renewable cellulose from lignocellulosic biomass toward functional products. J Clean Prod 414:137708. doi:10.1016/j.jclepro.2023.137708

[2] Liu G, Qu Y. 2021. Integrated engineering of enzymes and microorganisms for improving the efficiency of industrial lignocellulose deconstruction. Eng Microbiol 1:100005. doi:10.1016/j.engmic.2021.100005 39629162 PMC11610957

[3] Bischof RH, Ramoni J, Seiboth B. 2016. Cellulases and beyond: the first 70 years of the enzyme producer Trichoderma reesei. Microb Cell Fact 15:106. doi:10.1186/s12934-016-0507-6 27287427 PMC4902900

[4] Bayer EA, Belaich JP, Shoham Y, Lamed R. 2004. The cellulosomes: multienzyme machines for degradation of plant cell wall polysaccharides. Annu Rev Microbiol 58:521–554. doi:10.1146/annurev.micro.57.030502.091022 15487947

[5] Artzi L, Bayer EA, Moraïs S. 2017. Cellulosomes: bacterial nanomachines for dismantling plant polysaccharides. Nat Rev Microbiol 15:83–95. doi:10.1038/nrmicro.2016.164 27941816

[6] Xie G, Bruce DC, Challacombe JF, Chertkov O, Detter JC, Gilna P, Han CS, Lucas S, Misra M, Myers GL, Richardson P, Tapia R, Thayer N, Thompson LS, Brettin TS, Henrissat B, Wilson DB, McBride MJ. 2007. Genome sequence of the cellulolytic gliding bacterium Cytophaga hutchinsonii. Appl Environ Microbiol 73:3536–3546. doi:10.1128/AEM.00225-07 17400776 PMC1932680

[7] Taillefer M, Arntzen MØ, Henrissat B, Pope PB, Larsbrink J. 2018. Proteomic dissection of the cellulolytic machineries used by soil-dwelling Bacteroidetes. mSystems 3:e00240-18. doi:10.1128/mSystems.00240-18 30505945 PMC6247017

[8] Stanier RY. 1942. THE cytophaga group: a contribution to the biology of myxobacteria. Bacteriol Rev 6:143–196. doi:10.1128/br.6.3.143-196.1942 16350082 PMC440862

[9] McKee LS, La Rosa SL, Westereng B, Eijsink VG, Pope PB, Larsbrink J. 2021. Polysaccharide degradation by the Bacteroidetes: mechanisms and nomenclature. Environ Microbiol Rep 13:559–581. doi:10.1111/1758-2229.12980 34036727

[10] Foley MH, Cockburn DW, Koropatkin NM. 2016. The Sus operon: a model system for starch uptake by the human gut Bacteroidetes. Cell Mol Life Sci 73:2603–2617. doi:10.1007/s00018-016-2242-x 27137179 PMC4924478

[11] Grondin JM, Tamura K, Déjean G, Abbott DW, Brumer H. 2017. Polysaccharide utilization loci: fueling microbial communities. J Bacteriol 199:e00860-16. doi:10.1128/JB.00860-16 28138099 PMC5512228

[12] Tancula E, Feldhaus MJ, Bedzyk LA, Salyers AA. 1992. Location and characterization of genes involved in binding of starch to the surface of Bacteroides thetaiotaomicron. J Bacteriol 174:5609–5616. doi:10.1128/jb.174.17.5609-5616.1992 1512196 PMC206506

[13] Martens EC, Koropatkin NM, Smith TJ, Gordon JI. 2009. Complex glycan catabolism by the human gut microbiota: the Bacteroidetes Sus-like paradigm. J Biol Chem 284:24673–24677. doi:10.1074/jbc.R109.022848 19553672 PMC2757170

[14] Briggs JA, Grondin JM, Brumer H. 2021. Communal living: glycan utilization by the human gut microbiota. Environ Microbiol 23:15–35. doi:10.1111/1462-2920.15317 33185970

[15] Glenwright AJ, Pothula KR, Bhamidimarri SP, Chorev DS, Baslé A, Firbank SJ, Zheng H, Robinson CV, Winterhalter M, Kleinekathöfer U, Bolam DN, van den Berg B. 2017. Structural basis for nutrient acquisition by dominant members of the human gut microbiota. Nature 541:407–411. doi:10.1038/nature20828 28077872 PMC5497811

[16] Tamura K, Dejean G, Van Petegem F, Brumer H. 2021. Distinct protein architectures mediate species-specific beta-glucan binding and metabolism in the human gut microbiota. J Biol Chem 296:100415. doi:10.1016/j.jbc.2021.100415 33587952 PMC7974029

[17] Tauzin AS, Kwiatkowski KJ, Orlovsky NI, Smith CJ, Creagh AL, Haynes CA, Wawrzak Z, Brumer H, Koropatkin NM. 2016. Molecular dissection of xyloglucan recognition in a prominent human gut symbiont. MBio 7:e02134-15. doi:10.1128/mBio.02134-15 27118585 PMC4850273

[18] Grondin JM, Déjean G, Van Petegem F, Brumer H. 2022. Cell surface xyloglucan recognition and hydrolysis by the human gut commensal Bacteroides uniformis. Appl Environ Microbiol 88:e0156621. doi:10.1128/AEM.01566-21 34731054 PMC8752140

[19] Foley MH, Martens EC, Koropatkin NM. 2018. SusE facilitates starch uptake independent of starch binding in B. thetaiotaomicron. Mol Microbiol 108:551–566. doi:10.1111/mmi.13949 29528148 PMC5980745

[20] Tamura K, Foley MH, Gardill BR, Dejean G, Schnizlein M, Bahr CME, Louise Creagh A, van Petegem F, Koropatkin NM, Brumer H. 2019. Surface glycan-binding proteins are essential for cereal beta-glucan utilization by the human gut symbiont Bacteroides ovatus. Cell Mol Life Sci 76:4319–4340. doi:10.1007/s00018-019-03115-3 31062073 PMC6810844

[21] Cartmell A, Lowe EC, Baslé A, Firbank SJ, Ndeh DA, Murray H, Terrapon N, Lombard V, Henrissat B, Turnbull JE, Czjzek M, Gilbert HJ, Bolam DN. 2017. How members of the human gut microbiota overcome the sulfation problem posed by glycosaminoglycans. Proc Natl Acad Sci U S A 114:7037–7042. doi:10.1073/pnas.1704367114 28630303 PMC5502631

[22] Zhu Y, Kwiatkowski KJ, Yang T, Kharade SS, Bahr CM, Koropatkin NM, Liu W, McBride MJ. 2015. Outer membrane proteins related to SusC and SusD are not required for Cytophaga hutchinsonii cellulose utilization. Appl Microbiol Biotechnol 99:6339–6350. doi:10.1007/s00253-015-6555-8 25846333

[23] Zhou H, Wang X, Yang T, Zhang W, Chen G, Liu W. 2016. An outer membrane protein involved in the uptake of glucose is essential for Cytophaga hutchinsonii cellulose utilization. Appl Environ Microbiol 82:1933–1944. doi:10.1128/AEM.03939-15 26773084 PMC4784033

[24] Gao L, Su Y, Song W, Zhang W, Qi Q, Lu X. 2022. A type IX secretion system substrate involved in crystalline cellulose degradation by affecting crucial cellulose binding proteins in Cytophaga hutchinsonii. Appl Environ Microbiol 88:e0183721. doi:10.1128/AEM.01837-21 34731049 PMC8788686

[25] Zhang C, Zhang W, Lu X. 2015. Expression and characteristics of a Ca^2+^-dependent endoglucanase from Cytophaga hutchinsonii. Appl Microbiol Biotechnol 99:9617–9623. doi:10.1007/s00253-015-6746-3 26169628

[26] Ji X, Wang Y, Zhang C, Bai X, Zhang W, Lu X. 2014. Novel outer membrane protein involved in cellulose and cellooligosaccharide degradation by Cytophaga hutchinsonii. Appl Environ Microbiol 80:4511–4518. doi:10.1128/AEM.00687-14 24837387 PMC4148786

[27] Zhu Y, Han L, Hefferon KL, Silvaggi NR, Wilson DB, McBride MJ. 2016. Periplasmic Cytophaga hutchinsonii endoglucanases are required for use of crystalline cellulose as the sole source of carbon and energy. Appl Environ Microbiol 82:4835–4845. doi:10.1128/AEM.01298-16 27260354 PMC4984284

[28] Wang X, Zhang W, Zhou H, Chen G, Liu W. 2019. An extracytoplasmic function sigma factor controls cellulose utilization by regulating the expression of an outer membrane protein in Cytophaga hutchinsonii. Appl Environ Microbiol 85:e02606-18. doi:10.1128/AEM.02606-18 30578269 PMC6384103

[29] Blanvillain S, Meyer D, Boulanger A, Lautier M, Guynet C, Denancé N, Vasse J, Lauber E, Arlat M. 2007. Plant carbohydrate scavenging through tonB-dependent receptors: a feature shared by phytopathogenic and aquatic bacteria. PLoS One 2:e224. doi:10.1371/journal.pone.0000224 17311090 PMC1790865

[30] Déjean G, Blanvillain-Baufumé S, Boulanger A, Darrasse A, de Bernonville TD, Girard A-L, Carrére S, Jamet S, Zischek C, Lautier M, Solé M, Büttner D, Jacques M-A, Lauber E, Arlat M. 2013. The xylan utilization system of the plant pathogen Xanthomonas campestris pv campestris controls epiphytic life and reveals common features with oligotrophic bacteria and animal gut symbionts. New Phytol 198:899–915. doi:10.1111/nph.12187 23442088

[31] Greenfield NJ. 2006. Using circular dichroism collected as a function of temperature to determine the thermodynamics of protein unfolding and binding interactions. Nat Protoc 1:2527–2535. doi:10.1038/nprot.2006.204 17406506 PMC2752288

[32] Andersen N, Johansen KS, Michelsen M, Stenby EH, Krogh K, Olsson L. 2008. Hydrolysis of cellulose using mono-component enzymes shows synergy during hydrolysis of phosphoric acid swollen cellulose (PASC), but competition on Avicel. Enzyme Microb Technol 42:362–370. doi:10.1016/j.enzmictec.2007.11.018

[33] Wu ML, Chuang YC, Chen JP, Chen CS, Chang MC. 2001. Identification and characterization of the three chitin-binding domains within the multidomain chitinase Chi92 from Aeromonas hydrophila JP101. Appl Environ Microbiol 67:5100–5106. doi:10.1128/AEM.67.11.5100-5106.2001 11679332 PMC93277

[34] Sainz-Polo MA, González B, Menéndez M, Pastor FIJ, Sanz-Aparicio J. 2015. Exploring multimodularity in plant cell wall deconstruction: structural and functional analysis of xyn10c containing the CBM22-1-CBM22-2 tandem. J Biol Chem 290:17116–17130. doi:10.1074/jbc.M115.659300 26001782 PMC4498050

[35] Cameron EA, Kwiatkowski KJ, Lee BH, Hamaker BR, Koropatkin NM, Martens EC. 2014. Multifunctional nutrient-binding proteins adapt human symbiotic bacteria for glycan competition in the gut by separately promoting enhanced sensing and catalysis. MBio 5:e01441-14. doi:10.1128/mBio.01441-14 25205092 PMC4173775

[36] Lu X, Zhu Y, Zhang W. 2014. The family Cytophagaceae. Springer New York.

[37] Zhu YT, Thomas F, Larocque R, Li N, Duffieux D, Cladiere L, Souchaud F, Michel G, McBride MJ. 2017. Genetic analyses unravel the crucial role of a horizontally acquired alginate lyase for brown algal biomass degradation by Zobellia galactanivorans. Environ Microbiol 19:2164–2181. doi:10.1111/1462-2920.13699 28205313

[38] Zhu YT, Zhou H, Bi YL, Zhang WX, Chen GJ, Liu WF. 2013. Characterization of a family 5 glycoside hydrolase isolated from the outer membrane of cellulolytic Cytophaga hutchinsonii. Appl Microbiol Biotechnol 97:3925–3937. doi:10.1007/s00253-012-4259-x 22790541

[39] Bryksin AV, Matsumura I. 2010. Overlap extension PCR cloning: a simple and reliable way to create recombinant plasmids. Biotechniques 48:463–465. doi:10.2144/000113418 20569222 PMC3121328

[40] Zhu YT, Li HH, Zhou H, Chen GJ, Liu WF. 2010. Cellulose and cellodextrin utilization by the cellulolytic bacterium Cytophaga hutchisonii. Bioresour Technol 101:6432–6437. doi:10.1016/j.biortech.2010.03.041 20362433

[41] Zhou H, Wang X, Yang T, Zhang W, Chen G, Liu W. 2015. Identification and characterization of a novel locus in Cytophaga hutchinsonii involved in colony spreading and cellulose digestion. Appl Microbiol Biotechnol 99:4321–4331. doi:10.1007/s00253-015-6412-9 25661809

[42] Wang S, Zhao D, Bai X, Zhang W, Lu X. 2017. Identification and characterization of a large protein essential for degradation of the crystalline region of cellulose by Cytophaga hutchinsonii. Appl Environ Microbiol 83:e02270-16. doi:10.1128/AEM.02270-16 27742681 PMC5165126

[43] Forsberg Z, Nelson CE, Dalhus B, Mekasha S, Loose JSM, Crouch LI, Røhr ÅK, Gardner JG, Eijsink VGH, Vaaje-Kolstad G. 2016. Structural and functional analysis of a lytic polysaccharide monooxygenase important for efficient utilization of chitin in Cellvibrio japonicus. J Biol Chem 291:7300–7312. doi:10.1074/jbc.M115.700161 26858252 PMC4817163

[44] Larkin MA, Blackshields G, Brown NP, Chenna R, McGettigan PA, McWilliam H, Valentin F, Wallace IM, Wilm A, Lopez R, Thompson JD, Gibson TJ, Higgins DG. 2007. Clustal W and Clustal X version 2.0. Bioinformatics 23:2947–2948. doi:10.1093/bioinformatics/btm404 17846036

[45] Söding J, Biegert A, Lupas AN. 2005. The HHpred interactive server for protein homology detection and structure prediction. Nucleic Acids Res 33:W244–W248. doi:10.1093/nar/gki408 15980461 PMC1160169

