# Supplementary material for: SGBP-B-like bimodular cellulose-binding protein CHU_1279 is essential for cellulose utilization by Cytophaga hutchinsonii
Source: Appl Environ Microbiol. 2025 Mar 25;91(4):e02471-24. doi: 10.1128/aem.02471-24 (PMC12016534; doi:10.1128/aem.02471-24)
Supplement: Supplemental figures — Figures S1 to S3. [file aem.02471-24-s0001.docx]

**Supplementary materials**

**A** **SGBP-B-like bimodular cellulose-binding protein CHU_1279 is essential for cellulose utilization by *Cytophaga hutchinsonii***

Weixin Zhang^1^, Lizhu Li^1^, Tengxin Li^1^, Xin Li^1^, Xia Wang^1^, Xuemei Lu^1^, Qiang Yao^2^, Guanjun Chen^1,^*, Weifeng Liu^1,^*

^1^State Key Laboratory of Microbial Technology, Shandong University, No.72 Binhai Road, Qingdao 266237, P. R. China

^2^National Center of Technology Innovation for Comprehensive Utilization of Saline-Alkali Land, No. 8 Zhihui Road, Agricultural high-tech industry demonstration zone, Yellow River Delta, Dongying 257347, P. R. China

^*^Correspondence should be addressed to Weifeng Liu, E-mail: [weifliu@sdu.edu.cn](mailto:weifliu@sdu.edu.cn); Guanjun Chen, guanjun@sdu.edu.cn


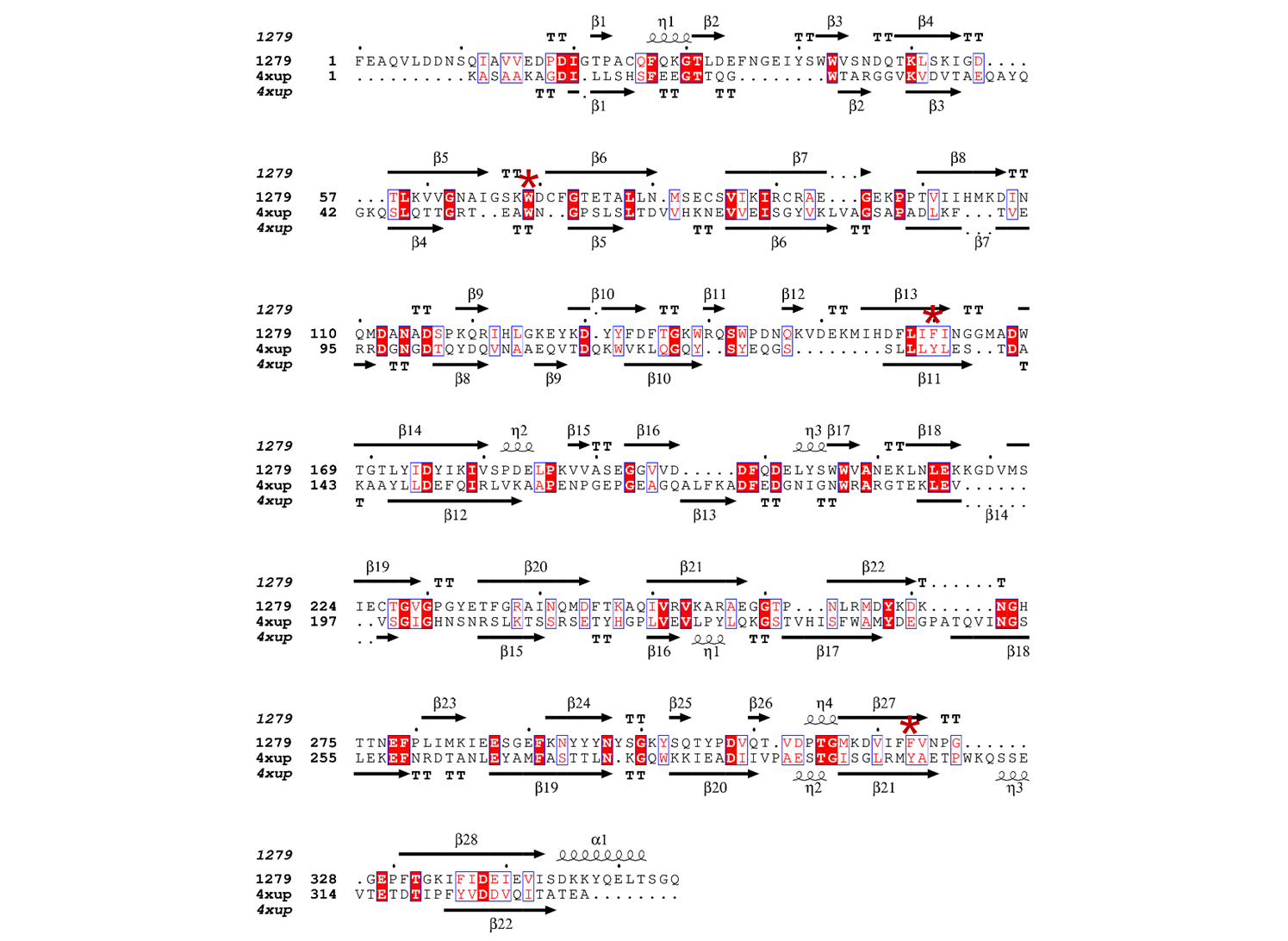


Figure S1 Sequence alignment of CHU_1279 excluded the putative signal peptide with the tandem of two CBMs in Xyn10C of *Paenibacillus barcinonensis* (PDB No. 4xup). CHU_1279 has equivalent residues identical or similar to three out of six aromatic residues involved in ligand binding in the CBM tandem in Xyn10C, namely Trp89, Phe180, and Phe343, which were indicated by red asterisks.


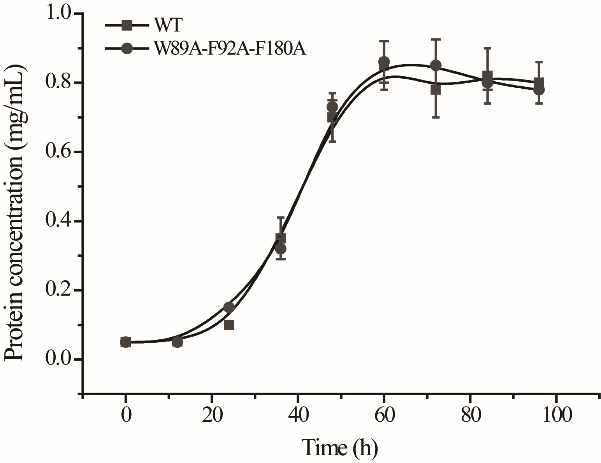


Figure S2 Growth analyses of ∆*chu_1279* expressing the WT CHU_1279 or a triple site-directed mutant (W89A-F92A-F180A) with Avicel as the sole carbon source.


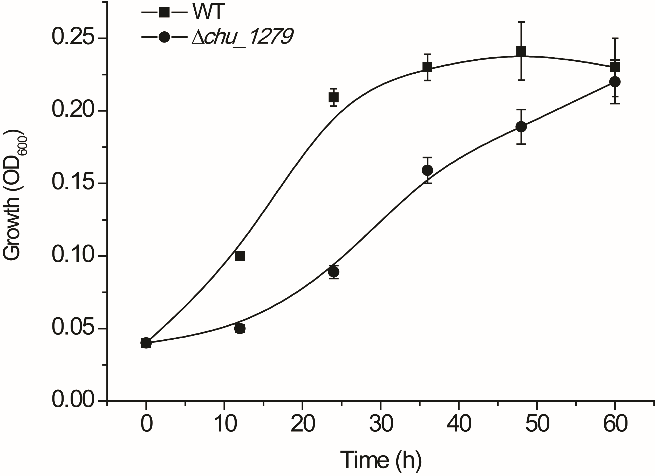


Figure S3 Growth analyses of WT and ∆*chu_1279* in liquid PY10 medium supplemented with 0.1% glucose.
